# Semi-automated histogram analysis of normal bone marrow using ^18^F-FDG PET/CT: correlation with clinical indicators

**DOI:** 10.1186/s12880-022-00757-x

**Published:** 2022-02-23

**Authors:** Yoko Satoh, Satoshi Funayama, Hiroshi Onishi, Keita Kirito

**Affiliations:** 1 Yamanashi PET Imaging Clinic, Shimokato 3046-2, Chuo City, Yamanashi Prefecture 409-3821 Japan; 2 Department of Radiology, University of Yamanashi, Shimokato 1110, Chuo City, Yamanashi Prefecture 409-3898 Japan; 3 Department of Hematology and Oncology, University of Yamanashi, Shimokato 1110, Chuo City, Yamanashi Prefecture 409-3898 Japan

**Keywords:** F-18 fluorodeoxyglucose positron emission tomography/computed tomography (FDG-PET/CT), Bone marrow, Semi-automatic extraction, Normal uptake pattern, Histogram analysis

## Abstract

**Background:**

^18^F-fluorodeoxyglucose (FDG) positron emission tomography (PET) is increasingly applied to the diagnosis of bone marrow failure such as myeloproliferative neoplasm, aplastic anemia, and myelodysplastic syndrome, as well as malignant lymphoma and multiple myeloma. However, few studies have shown a normal FDG uptake pattern. This study aimed to establish a standard of bone marrow FDG uptake by a reproducible quantitative method with fewer steps using deep learning-based organ segmentation.

**Methods:**

Bone marrow PET images were obtained using segmented whole-spine and pelvic bone marrow cavity CT as mask images using a commercially available imaging workstation that implemented an automatic organ segmentation algorithm based on deep learning. The correlation between clinical indicators and quantitative PET parameters, including histogram features, was evaluated.

**Results:**

A total of 98 healthy adults were analyzed. The volume of bone marrow PET extracted in men was significantly higher than that in women (*p* < 0.0001). Univariate and multivariate regression analyses showed that mean of standardized uptake value corrected by lean body mass (SUL_mean_) and entropy in both men and women were inversely correlated with age (all *p* < 0.0001), and SUL_max_ in women were also inversely correlated with age (*p* = 0.011).

**Conclusion:**

A normal FDG uptake pattern was demonstrated by simplified FDG PET/CT bone marrow quantification.

## Background

Whole-body ^18^F-fluorodeoxyglucose (FDG) positron emission tomography/computed tomography (PET/CT) is a glucose metabolism-based imaging technique that is currently used for staging, restaging, and therapeutic evaluation of malignant tumors. PET is a diagnostic imaging modality with excellent quantitative properties and the various quantitative values that are calculated based on the standardized uptake value (SUV), which is a semi-quantitative value of FDG accumulation, are useful for distinguishing benign and malignant tumors and predicting their prognosis [1–3].

In addition to malignant tumors, bone marrow (BM) FDG uptake could be affected by hematologic, immune, and inflammatory disorders, which cause increased cellularity and hyperactivity in the BM [4–7]. BM FDG uptake has also been reportedly associated with white blood cell (WBC) count, serum C-reactive protein, and granulocyte-colony stimulating factor or erythropoietin [8, 9]. Although BM biopsy remains the gold standard for BM pathology diagnosis in clinical practice, BM biopsies are highly invasive and contain many sampling errors. Particular attention should be paid to the irregular distribution of local areas of active bone marrow and/or hematological malignancies, and thus, the lesions may be missed on random biopsies.

FDG-PET/CT has enabled less invasive visualization of glucose metabolism in BM throughout the body, which was considerably difficult with conventional morphological imaging such as CT and magnetic resonance imaging [10]*.* Recently, FDG PET/CT has been increasingly applied to the diagnosis of bone marrow failure such as myeloproliferative neoplasm, aplastic anemia, and myelodysplastic syndrome as well as malignant lymphoma and multiple myeloma [11–14]. Some studies have reported that the region-of-interest was manually placed on certain bones and based on the semi-automatic measurement method for quantitative evaluation of BM [14, 15]. The segmentation of whole BM by a computational approach of PET/CT images has also been reported [16, 17]. However, the PET quantification method for BM, which was proposed in the previous studies, was complicated and has not been clinically applied. In addition, the quantitative values representing the FDG uptake pattern of normal bone marrow as a baseline have been undefined.

Therefore, the purpose of this study was to establish a standard for bone marrow FDG uptake patterns by a reproducible quantitative method with fewer steps using a commercially available automated image analysis software.

## Methods

### Participant enrollment

Between May 2017 and March 2020, 667 consecutively registered participants underwent whole-body PET/CT for cancer screening at our institution. The inclusion criteria were: (i) adults aged 20–80 years, (ii) no abnormalities detected on PET/CT, and absence of abnormalities confirmed on subsequent PET/CT within the following two years, and (iii) answered a questionnaire about medical history, abnormal symptoms, sex, age, and smoking history before cancer screening. Participants with a history of malignancies, systemic diseases (hematologic disorders, immune diseases, infection/inflammation, fever), and those receiving hormonal therapy, chemotherapy, immunosuppressive therapy, or steroids were excluded. Participants with diabetes and those with blood glucose levels ≥ 150 mg/dL before FDG injection were also excluded. However, there were no adults in their 20s who underwent PET for the purpose of cancer screening. It has been considered better to include those in their 20s in order to target "healthy adults". For that reason, four women in their 20s who had a PET scan for purposes other than health checks and were finally diagnosed as "healthy" were added. Unfortunately, no man in his twenties met these inclusion criteria.

Participants were excluded from this study if they had white blood cell (WBC) count, mean corpuscular volume, hemoglobin level, platelets count, lactic acid dehydrogenase, creatinine, aspartate aminotransferase, alanine aminotransferase, and total bilirubin levels in their blood data that are not in the normal range (Table 1). No participant had splenomegaly on CT [18]. All the participants were considered healthy without clinical suspicion of malignancy or a severe inflammatory condition based on the diagnosis by experienced physicians who performed medical interviews and physical examinations, and imaging diagnostic radiologists who diagnosed them comprehensively.

**Table 1:** Blood data items referenced for the definition of healthy adults in this study

Item	Abbreviation	Normal range	
White blood cell counts	WBC (× 10^3^/μL)		3.5–9.7
Hemoglobin level	Hb (g/dL)	Men	12.6–17.6
		Women	10.6–15.2
Mean Corpuscular Volume	MCV (fl)	Men	83.3–101.4
		Women	79.1–100.0
Platelet counts	Plt (× 10^4^/μL)		13.8.-37.9
Lactic acid dehydrogenase	LDH (IU/L)		110–220
Creatinine	Cre (mg/dL)	Men	≤ 1.0
		Women	≤ 0.7
Aspartate aminotransferase	AST (IU/L)		8–35
Alanine aminotransferase	ALT (IU/L)		5–30
Total bilirubin	T. Bil (mg/dL)		0.2–1.2

This single-institution study was approved by our institutional review board and was conducted in accordance with the Declaration of Helsinki. Owing to the retrospective study design and use of anonymized patient data, the requirement for informed consent was waived. We had the right to access the clinical/personal patient data used in this study and were not required to obtain any special administrative permissions or licenses.

### Imaging protocol

All the patients fasted for at least 6 h before ^18^F-FDG (3 MBq/kg) administration. Whole-body PET/CT scans were performed at 60-min post injection using a Biograph Horizon TrueV FDG-PET/CT system (Siemens Medical Solutions, Knoxville, TN, USA). The coincidence-timing window and time-of-flight system-timing resolution were 4.1 ns and 540 ps, respectively. A CT scan was performed for attenuation correction (tube voltage, 130 kV; tube current, 15–70 mA; tube rotation time, 0.6 s per rotation; pitch, 1; transaxial field of view [FOV], 700 mm; and section thickness, 5 mm). The PET/CT images were reconstructed using the ordered subset expectation–maximization method and time-of-flight algorithm with 4 iterations and 10 subsets. The CT data were resized from a 512 × 512 matrix to a 180 × 180 matrix to match the PET data and construct the CT-based transmission maps for attenuation correction of the PET data with a post-reconstruction smoothing Gaussian filter (5-mm FWHM). The reconstructed voxel size of the PET/CT images was 4.11 × 4.11 × 5 mm.

### Preparation of BM PET

The extraction of “BM PET” was planned in two major steps using a CT volume analyzer (SYNAPSE VINCENT medical imaging system; Fujifilm Medical, Tokyo, Japan) which implemented an algorithm for automatic bone segmentation based on deep learning [19, 20]. In order to run this image analyzer, it was necessary to install it on a computer with an operating system of Windows 7 or later versions, a Pentium 4 or more central processing unit, and memory of 1 GB or more.

**Fig. 1:**
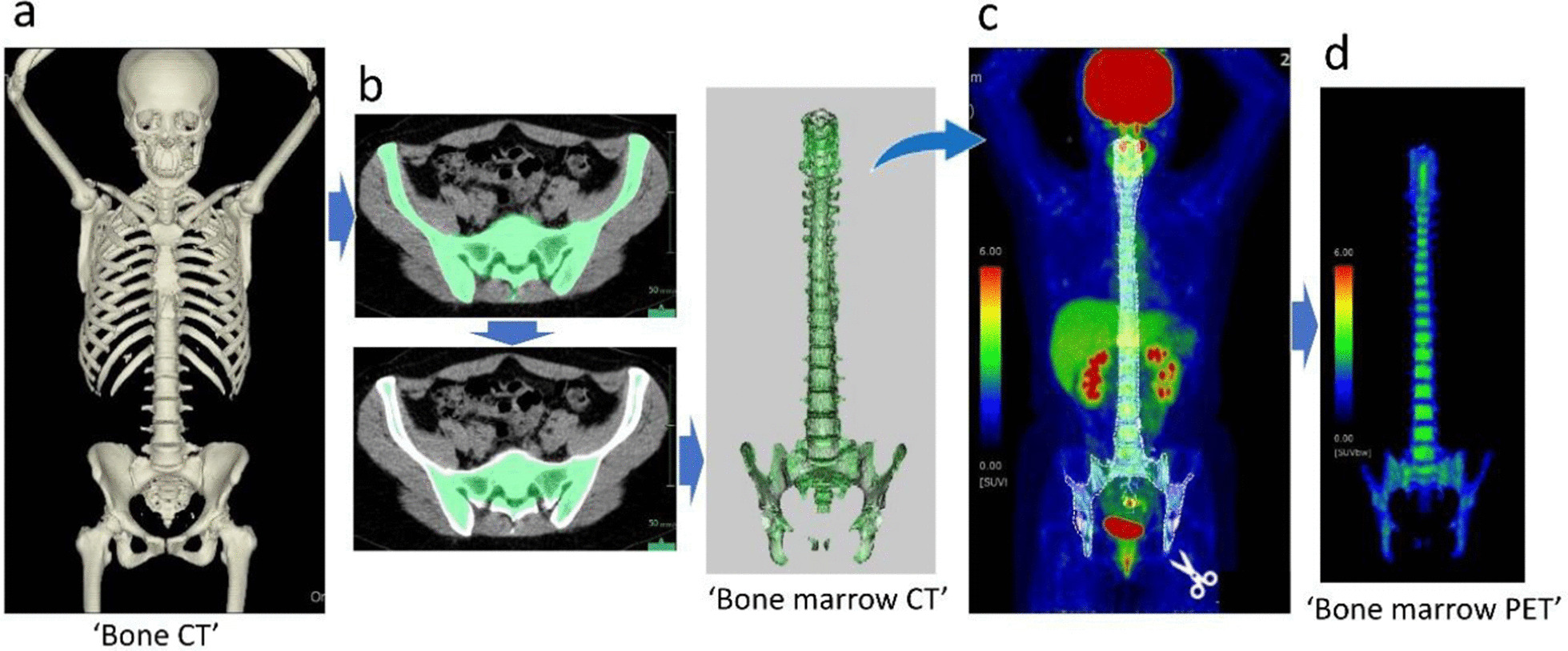
Two major procedures to obtain bone marrow (BM) positron emission tomography (PET). First: “BM computed tomography (CT)" was obtained. Whole spine and pelvic bones, including the BM, were automatically extracted from whole-body CT images using a bone-extraction algorithm implemented in the analyzer for automatic bone segmentation based on multi-task three-dimensional fully convolutional networks (3D-FCN) [19]. A multi-task 3D-FCN for organ segmentation has surpassed the two-dimensional FCN approach [20] by using data from multiple segmentation datasets and showed improvement over the single-task 3D-FCN approach (**a**). The contour lines were subsequently set to 2 pixels inside the first line, which were able to extract the BM and remove as much cortical bone as possible (**b**). Second: overlay bone marrow CT on whole-body PET (**c**), and obtain “bone marrow PET” masked with bone marrow CT (**d**)

**Fig. 2:**
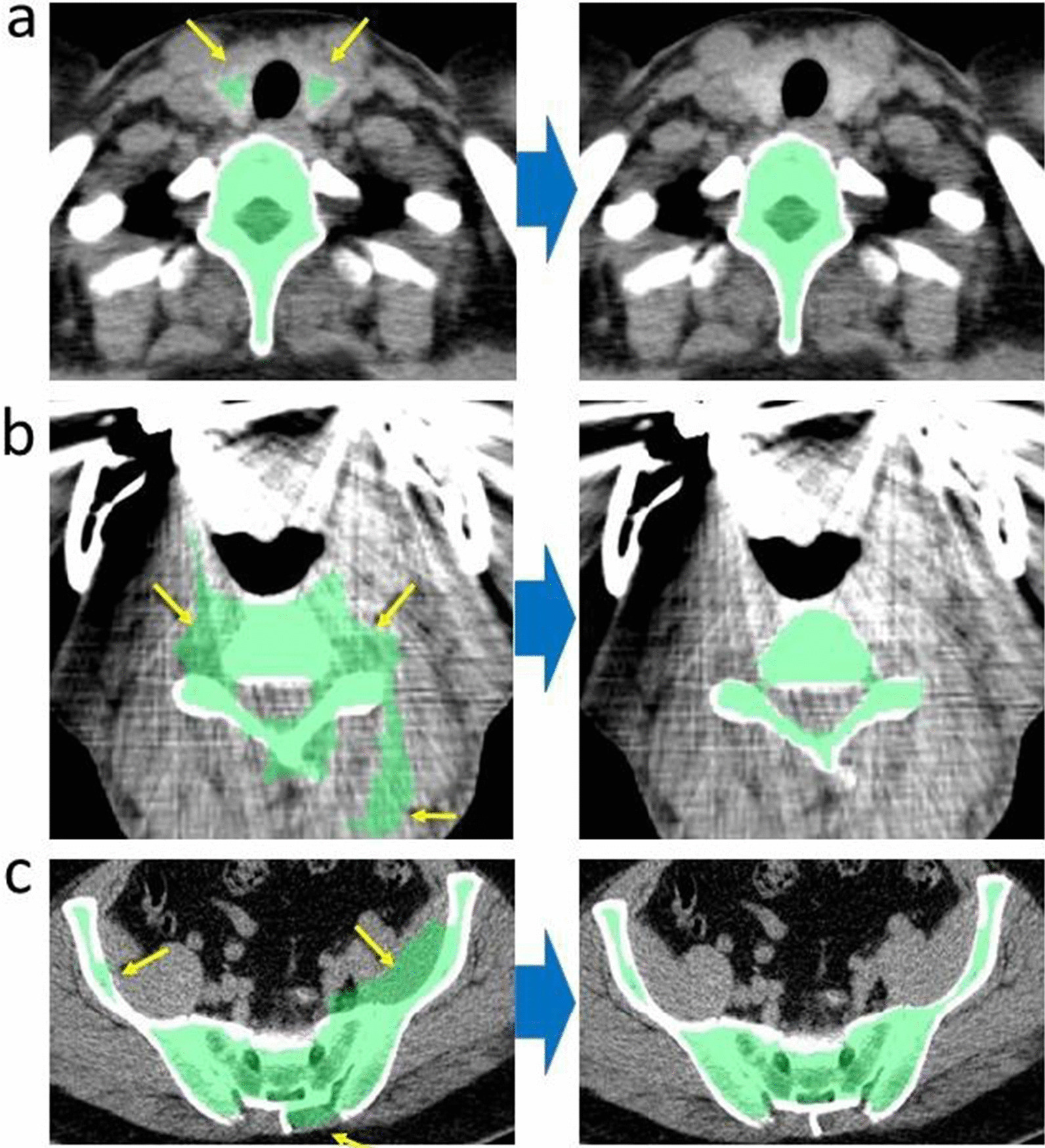
Addition of manual removal of the extracted extramedullary region due to high CT value, thyroid gland (**a**), metal for dental treatment (**b**). Images of a case where the pelvic muscles extracted continuously with the pelvic bones were manually removed (**c**). No obvious artifacts were visible

Figure 1 shows an overview of the process. In this study, the spine and pelvic bone marrows, which are the main components of hematopoiesis in disease-free adults, were segmented [15, 21]. The extremities were excluded in this study since these PET/CTs were performed for cancer screening in healthy adults, and skull was also excluded since it was affected by artifacts due to the high physiological FDG uptake of the brain. To summarize the "BM CT" segmentation procedure, first, on CT, all spinal bones were segmented using the automatic organ extraction algorithm for "whole spine" implemented in the volume analyzer. Separately, the pelvic bone was segmented using an algorithm for extracting certain designated bones and then added to the previously obtained total spinal bones. In addition, 2 pixels were removed from the margin to exclude the bone cortex. When extraosseous tissue was extracted due to a high CT value, metal, thyroid gland, among others, the relevant part was manually removed (Fig. 2). Second, "BM PET" was extracted from whole-body PET images using "BM CT" as a mask image. BM of the 11th thoracic vertebra (Th11) was extracted separately from the BM of the whole spine and pelvis with the same procedure for Deauville 5-point scaling.

A board-certified nuclear medicine physician performed a series of image processing procedures. To assess reproducibility, the same interpreter repeated these steps twice for all participants.

### Data analysis

SUV is often used as a PET parameter for the evaluation of solid tumors; however, the SUV corrected by lean body mass, referred to as SUL, was used in this study for an accurate evaluation of normal bone marrow with large volumes. This is because FDG was injected in proportion to the body weight, and although the body weight depends on fat, the uptake of FDG in fat is small. Lean body weight was defined according to a previous study [22].

After converting all voxel values in the BM PET image to SULs, the quantitative PET parameters of maximum SUV corrected by lean body mass (LBM) (SUL_max_), mean of SUL (SUL_mean_), and entropy, one of the features of the grayscale histogram, were calculated.

The SUL and LBM of a given tissue were calculated using the following formula [22]:

$$\begin{aligned} & {\text{SUL}} = \frac{{{\text{Tumor}}\;{\text{activity}}\;{\text{concentration }}\left( {{\text{Bq}}/{\text{ml}}} \right)}}{{{\text{Injected}}\;{\text{dose }}\left( {{\text{Bq}}} \right)}} \times {\text{LBM}}\;\left( {\text{g}} \right), \\ & {\text{LBM}}^{{{\text{man}}}} = 9270 \times \frac{{{\text{Body}}\;{\text{weight}}}}{{6680 + 216{ } \times {\text{ BMI}}}} , \\ & {\text{LBM}}^{{{\text{woman}}}} = 9270 \times \frac{{\text{Body weight}}}{{8780 + 244{ } \times {\text{ BMI}}}} . \\ \end{aligned}$$

The SUL_mean_ is the average value of extracted BM PET images.

In addition, the liver and the mediastinal SULs were measured using syngo.via VB10 software (Siemens Healthcare GmbH, Erlangen, Germany), which implemented an algorithm to automatically set an resion of interest on them for Deauville 5-point scaling to ensure objectivity and reproducibility. The two software (SYNAPSE VINCENT and syngo.via) have been tested and verified to display the same value using the same Digital Imaging and COmmunications in Medicine: DICOM storage information. Percentage of BM volume with higher SUL than that of mediastinum (% Higher volume) was calculated, and the Deauville score of Th11 BM for each object was determined according to the previous report [15].

Additionally, some clinical anthropometric and blood-related data including sex, age, LBM, smoking history, WBC count, and hemoglobin level were evaluated.

### Statistical analysis

To evaluate the confounding indicators between men and women, the difference between WBC and hemoglobin level (normally distributed) was compared using the t-test, and the differences between other clinical indicators were compared using the Mann–Whitney U test. Multivariate regression analysis was subsequently performed using the clinical indicators that significantly correlated with the PET-derived parameters in the previously performed univariate analyses. Regression analyses between continuous clinical indicators and PET derived parameters were performed using Pearson's correlation coefficient and between smoking habit and PET derived parameters using Spearman's correlation coefficient. In group comparison of the 5-point score and clinical indicators, differences were assessed using a one-way analysis of variance (ANOVA). Chi-square tests, or, for low frequencies, Fisher’s exact test, were used to test for differences between the 5-point scale and smoking habit. One reader performed the entire image processing process twice; the reproducibility of the BM PET extraction was evaluated by the intersection over union for pixel accuracy and the intraclass correlation coefficient for the SUL_mean_. The second procedure was performed at least 30 days following the first analysis, with the order randomly changed. Statistical significance was set at *p* < 0.05. All analyses were performed using the JMP®15 (SAS Institute Inc., Cary, NC, USA) statistical software.

## Results

A total of 98 healthy adults (57 men and 41 women) were enrolled in this study (Table 2). The study participants included 5 (5 women), 18 (12 men, 6 women), 12 (8 men, 4 women), 15 (11 men, 4 women), 25 (14 men, 11 women), 16 (8 men and 8 women), and 7 (3 men and 4 women) participants in their 20 s, 30 s, 40 s, 50 s, 60 s, 70 s, and 80 s, respectively. The average and standard deviation of age was 55.5 ± 14.2 and 56.3 ± 18.9 for men and women, respectively, with no significant difference. The average LBM, and hemoglobin level were significantly different between men and women (*p* < 0.0001, Table 2). These results indicated that sex was a covariate, and the following univariate and multivariate analyses were performed for each group of men and women.

**Table 2:** Characteristics of healthy participants

	Men n = 57	Women n = 41	*p*
**Age (y)**			
Average ± SD	55.5 ± 14.2	56.3 ± 18.9	0.637
**LBM**			
Average ± SD	52.6 ± 6	34.7 ± 5.4	< 0.0001*
**WBC (× 10**^**3**^**/μL)**			
Average ± SD	6 ± 1.6	5.3 ± 1.1	0.0758
**Hb (g/dL)**			
Average ± SD	15 ± 1.1	13 ± 0.9	< 0.0001*
**Smoking habit**			
Current	17 (30%)	5 (12%)	0.0502
Former†/non	40 (70%)	36 (88%)	

*Statistically significant. †Who was a smoker more than five years ago and has not smoked for the last five years

*SD* standard deviation, *LBM* lean body mass, *WBC* white blood cell count; Hb, hemoglobin

Extraction of BM PET from one subject took about 3 min with the automated process alone, and was completed within 5 min even if manual correction was required. The intersection over union for the extracted pixel accuracy and the intraclass correlation coefficient for the SUL_mean_ was conducted for the groups of men and women. The extracted BM PET had a mean intersection over union of 0.99, and a SUL_mean_ intraclass correlation coefficient of 0.99, indicating an excellent reproducibility. In 70 of the 98 cases (71.4%), the BM PET was completely extracted automatically. In the other 28 cases (28.6%) that required manual correction (due to high CT values of thyroids: 7, metals for dental treatment: 9, pelvic arterial calcifications: 4, artifacts of pelvic bone: 4, inseparable pelvic and femur: 2, metal for lumbar spine surgery:1, and metal for femur surgery:1), the mean and standard deviation of intersection over union were 0.9875 and 0.0245, respectively. Among the BM PET-derived parameters, the volume, %Higher volume, and Deauville 5-point score were significantly different between men and women (Table 3). The volume increased with increase in LBM in women (r = 0.39, *p* = 0.0108).

**Table 3:** Comparison of bone marrow quantitative positron emission tomography (PET) parameters between men and women

	Men n = 57	Women n = 41	*p*
Volume (cm^3^)	1140.24 ± 154.75	798.38 ± 104.62	< 0.0001*
SUL_max_	2 ± 0.38	1.73 ± 0.28	0.0001*
SUL_mean_	0.79 ± 0.1	0.75 ± 0.11	0.024*
Entropy	4.38 ± 0.19	4.19 ± 0.23	< 0.0001*
%Higher volume	3.87 ± 0.73	2.15 ± 0.43	0.0063*
**Deauville 5-point score**^**†**^			
2	31	7	0.0002*
3	23	25	
4	3	9	

SUL_max_, maximum standard-uptake value corrected by lean body mass; SUL_mean_, mean standard-uptake value corrected by lean body mass; %Higher volume, percentage of bone marrow volume with higher SUL than that of mediastinum

*Statistically significant. The numbers in the table show the mean ± standard deviation. †SUL_mean_ of bone marrow of the 11th thoracic spine: 2 = below the mediastinum, 3 = above the mediastinum and below the liver, 4 = above that of the liver

### Correlation between clinical indicators and PET-derived parameters

The results of the univariate analyses between the clinical indicators and PET parameters are shown in Table 4. Age was associated with SUL_mean_ and entropy in both men and women and with SUL_max_ in women. Hemoglobin level was associated with SUL_mean_ in men. In a multivariate analysis of SUL_mean_ in men, only age was significantly correlated.

**Table 4:** Univariate analyses between clinical indicators and positron emission tomography (PET)-derived parameters

	Men n = 57				Women n = 41			
	Age	WBC	Hb	Current smoker (−/ +)	Age	WBC	Hb	Current smoker (−/ +)
**SUL**_**max**_								
r	−0.1028	0.26	0.13		−0.3929	0.0849	0.2894	
* p*	0.4465	0.0508	0.9734	0.2356	0.0110^*^	0.5977	0.0665	0.1632
**SUL**_**mean**_								
r	−0.541	0.2145	0.3041		−0.8097	0.1351	0.2733	
* p*	< 0.0001^*^	0.109	0.0215^*^	0.6008	< 0.0001^*^	0.3966	0.0839	0.2168
**Entropy**								
r	−0.5279	0.1136	0.0855		−0.6541	0.0205	0.1087	
* p*	< 0.0001^*^	0.4002	0.5271	0.9861	< 0.0001^*^	0.8989	0.4987	0.1202
**% Higher volume**								
r	−0.259	0.0752	0.0666		−0.3994	−0.1098	0.1527	
* p*	0.0517	0.5783	0.6227	0.5187	0.0097*	0.4944	0.3405	0.5187
**Deauville 5-point score**^**†**^								
* p*	0.2524	0.5485	0.7451	0.3017	0.9959	0.3373	0.6931	0.2618

*WBC* white blood cell, *Hb* hemoglobin, *SUL*_*max*_ maximum standard uptake value corrected by lean body mass, *SUL*_*mean*_ mean standard-uptake value corrected by lean body mass, *%Higher volume* percentage of bone marrow volume with higher SUL than that of mediastinum

r = Pearson’s correlation coefficient (Spearman’s correlation coefficient for the smoking habit). A positive or negative *r* represents the direction of direct or inverse correlation, respectively

^†^Fisher's exact test

*Statistically significant. †SUL_mean_ of bone marrow of the 11th thoracic spine: 2 = below the mediastinum, 3 = above the mediastinum and below the liver, 4 = above that of the liver. “Current smoker” includes those who are smoking or previous smokers less than 5 years

**Fig. 3:**
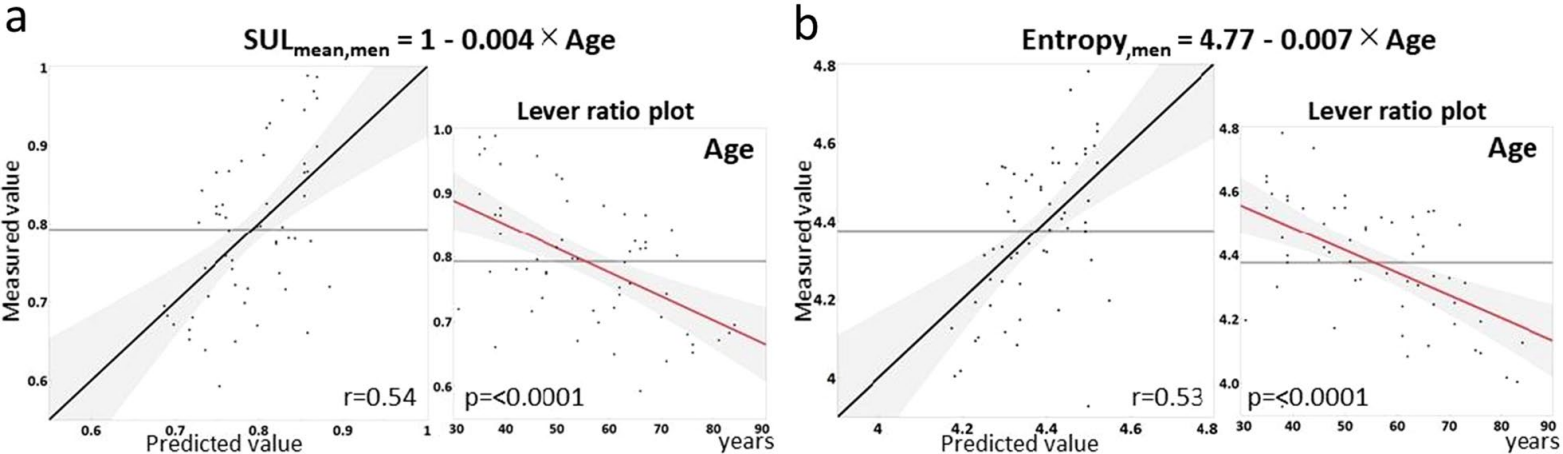
Prediction for mean standardized uptake value corrected by lean body mass (SUL_mean_) (**a**) and entropy (**b**) in men

**Fig. 4:**
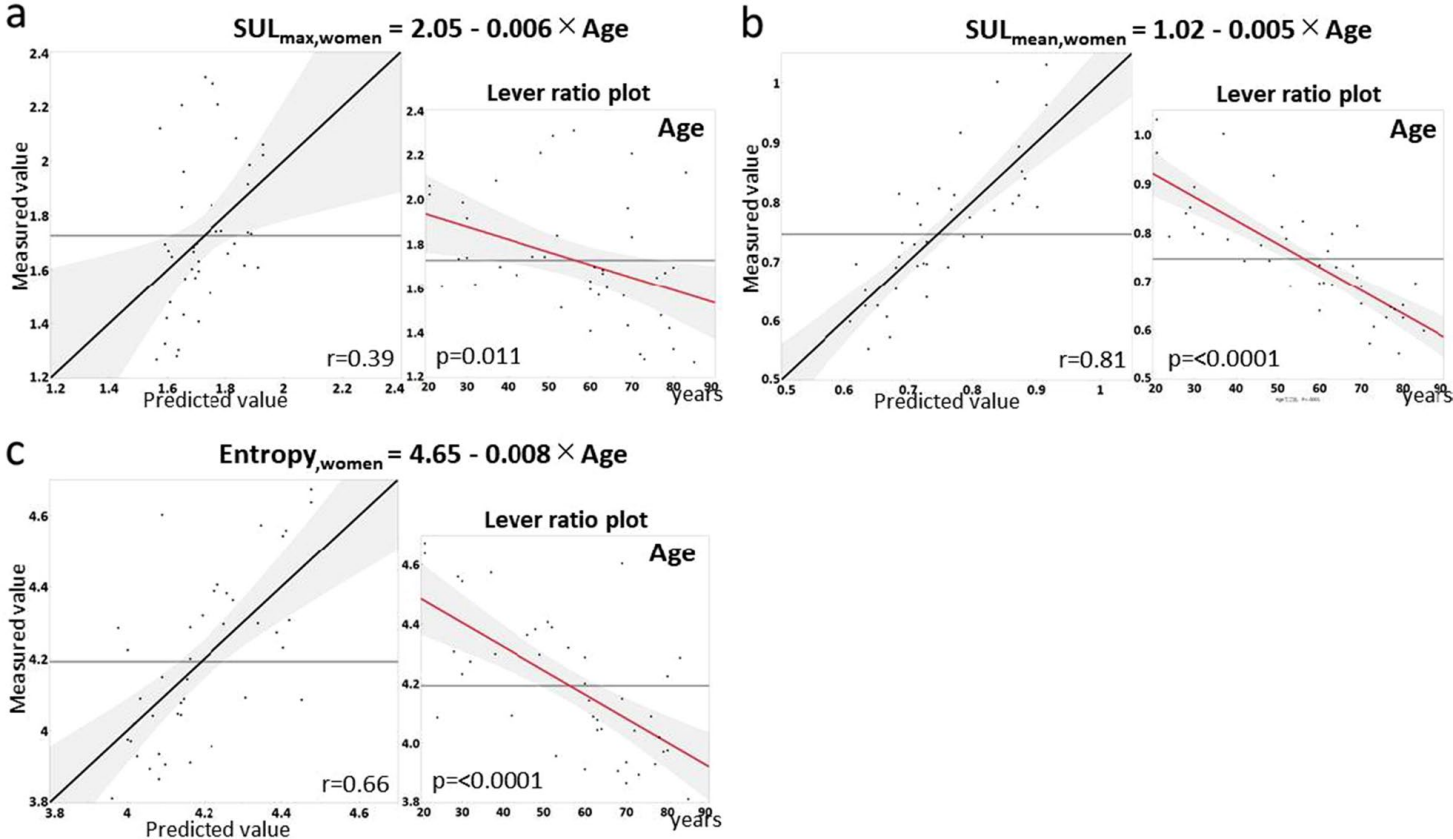
Prediction for maximum standardized uptake value corrected by lean body mass (SUL_max_) (**a**), mean standardized uptake value (SUL_mean_) (**b**) and entropy (**c**) in women

The measured and predicted values, prediction formulas, and lever ratio plots are shown in Fig. 3 and 4, where *r* represents the correlation coefficient.

**Fig. 5:**
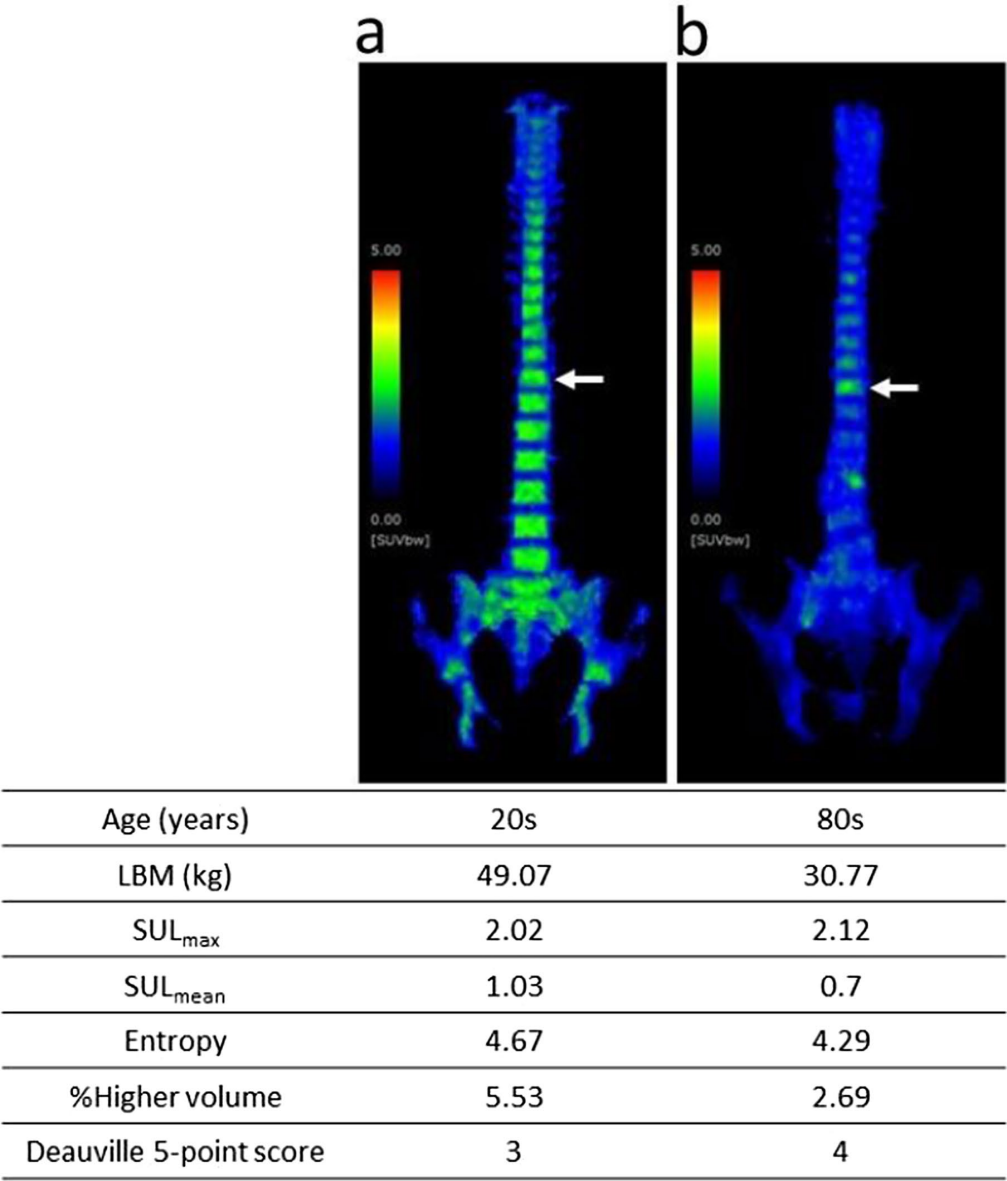
Maximum intensity projection image and bone marrow positron emission tomography (PET)-derived parameters of two women. FDG uptake in BM of a younger woman (**a**) was higher both visually and quantitatively (SUL_mean_ and % Higher volume) than that of a older woman (**b**). SUL, standardized uptake value correlated by lean body mass (LBM). White arrows indicate the 11th thoracic vertebral bodies

The representative cases are shown with maximum intensity projection images and the measured PET-derived parameters in Fig. 5. The BM FDG uptake was lower with age and increased with higher LBM.

## Discussion

Although the physiological uptake of FDG in the spinal BM has been shown to vary depending on the location of the vertebrae [15], to reduce intra- and interobserver variabilities, the BM extraction of all vertebrae and pelvis was calculated as one organ in this study. Extracted BM images of PET, which almost corresponded to the medullary cavities of the spine and pelvis, were automatically extracted using CT and PET images, and various PET quantitative values were measured. The FDG uptake in BM computed in our study was lower than that in previous reports, even with the difference that our method was corrected for LBM [15, 23] for both sexes. This may be due to background subjective variations and the measurement of larger volumes of BM of areas with low FDG uptake (upper cervical spine, sacrum, and pelvic bone) in the present study, whereas previous reports measured certain bones.

SUL_max_, SUL_mean_, entropy, and % higher volume of men were higher than those of women, and the proportion of women with a high Deauville score was higher than that of men. These results suggest that although the glucose metabolism of the BM of women is lower than that of men, the bone marrow of women has a relatively high glucose metabolism than other organs. This may be due to the difference in body size between men and women and the presence or absence of menstruation. Since the distribution of normal bone marrow cells is heterogeneous compared to abnormal increases in cell density such as tumors, differences in entropy between men and women may be the same reason. Furthermore, our study showed that FDG uptake in the BM decreased with age in both men and women, it was consistent with the previous studies [21, 23]. Since all of the participants' WBC and hemoglobin levels were in the normal range, the effects of abnormally low and high levels of these hematological indicators on BM uptake could not be fully elucidated. Furthermore, the entropy was also evaluated as one of the histogram features computed from BM PET images. The entropy of BM PET was higher with younger age, indicating heterogeneity in both men and women. Patient-based studies on these findings are warranted.

van Son et al. reviewed previous reports on FDG uptake levels and patterns on FDG PET/CT of hemato-oncological conditions [24]. The review summarized that inflammation and colony-stimulating factors caused a diffuse FDG uptake in the bone marrow that was higher than the physiological FDG uptake in the liver. Diffuse FDG uptake in the BM has also been found in myeloproliferative neoplasms [11, 12], while hyperplastic hematopoietic islands in aplastic anemia showed island-like and localized FDG uptake [13, 25], and BM reconversion with island-like and localized FDG accumulation following the treatment of malignant tumors were observed. Although multiple myeloma had a relatively low diffuse uptake in BM, the CT often showed bone abnormalities such as osteolytic mass and fracture [24]. Bone marrow invasion of malignant lymphoma often presented with localized high uptakes and might also present with diffuse uptakes [24]. Although these findings greatly influence the diagnosis and follow-up of various diseases, quantitative methods have not been established. Therefore, the FDG-uptake pattern in the BM could be characterized by the histogram parameters demonstrated in this study, which represent the FDG uptake’s heterogeneity. The FDG-uptake pattern obtained from this analysis serves as a baseline for future analysis of BM in various hematopoietic disorders.

In this study, we used a commercially available imaging workstation that implemented a recently developed deep learning-based organ segmentation technique for medical imaging [19, 26]. It achieved a highly reproducible and easy extraction of BM PET images of the spine and pelvis from PET/CT images. Although some previously reported computational approaches for bone and BM segmentation from CT-based PET/CT images exist, no direct comparison between these methods and ours was made [16, 17, 27]. Similar to the SUV measurement of malignant solid tumors being widespread in the clinical application of PET, standardization of a simple and accurate quantification method of BM PET for hematopoietic disorders is also necessary.

The major problem of this paper was that there was an insufficient assessment of the mechanism that brings the results of each parameter for each sex. This study evaluated only the bone marrow of healthy adults. The mechanism of various FDG uptake patterns in the bone marrow and its PET parameters can be elucidated by a comparative analysis of abnormal bone marrow accumulation caused by various diseases in the future.

Recent studies have revealed the feasibility of thymidine analog ^18^F-fluorothymidine (FLT) PET for the diagnosis and monitoring of BM disorders [28, 29]. FLT is a surrogate marker for DNA synthesis and is an excellent PET tracer that can clearly visualize the proliferative activity of the BM; however, it is still not available for other purposes excluding research. Application of the BM quantification method presented in this study to FLT-PET in the future could lead to more useful PET examinations for various hematopoietic disorders.

Our study had some limitations. First, there may have been a selection bias because this was a cross-sectional study using real-world PET imaging data for cancer screening. In addition, the sample size of the survey was small, with less than five participants in their twenties. In addition, some blood data containing C-reactive protein, which is known to correlate with BM-FDG uptake, were not analyzed. Therefore, more participants need to be tested. The blood test did not include CRP or erythrocyte sedimentation rate, which are indicators of inflammation, because it was associated with cancer screening of asymptomatic participants. However, these inflammatory indicators are associated with various diseases and should be included as routine tests. Second, the extracted BM PET contained some bone tissue, and it was not possible to prove that it completely matched the medullary cavity for each individual. Although 2 pixels were removed from the margin on bone CT, the removal of the cortical bone was not perfect because the thickness of the cortical bone could vary by site, age, and sex. However, FDG accumulation in normal bone (rather than bone marrow) is extremely low that it seems that glucose metabolism imaging of bone marrow could be represented almost accurately. In addition, if the area of the spinal canal surrounded by bone was small, it could not be excluded, as shown in Fig. 2a. However, if it was large, it could be excluded, as shown in Fig. 2b. In this study, we did not add manual corrections to establish a simpler method of bone marrow extraction. In the future, it will be improved by applying an algorithm that can accurately extract bone by CT value. Third, the extraction method was not completely automatic and some artifacts were manually removed. This will cause poor reproducibility. CTs reconstructed using the metal artifact reduction algorithm will be useful for fully automated extraction in future studies. Fourth, displacement of PET and CT images due to body movements during PET/CT scans is a major concern. Correction of the PET/CT fusion images using artificial intelligence, which has progressed remarkably, could be possible in the near future.

## Conclusions

We presented a simplified FDG-PET/CT BM quantification method and revealed uptake patterns in healthy adults using a histogram analysis. The SUL_mean_ and entropy were correlated with age in both men and women, and SUL_max_ was also correlated with age in women. These results could provide baseline findings for various BM abnormalities.

## Data Availability

The datasets generated and analyzed during the current study are not publicly available due to the security of data but are available from the corresponding author on reasonable request.

## Abbreviations

FDG: ^18^F-fluorodeoxyglucose
PET: Positron emission tomography
PET/CT: Positron emission tomography/computed tomography
SUL: Standardized uptake value corrected by lean body mass (LBM)
BM: Bone marrow
WBC: White blood cell count
FLT: ^18^F-fluorothymidine
